# Analysis of perinatal outcomes for emergency cervical cerclage in singleton pregnancies at 24–28 weeks of gestation

**DOI:** 10.1007/s00404-024-07493-3

**Published:** 2024-04-23

**Authors:** Xiling Yi, Dan Zhang, Jing Yang, Hongyan Gao, Hengyu Cai, Jianping Cong, Chengli Lin

**Affiliations:** Department of Obstetrics and Gynaecology, Shenyang Women’s and Children’s Hospital, Shenyang, 110000 Liaoning China

**Keywords:** 24–28 weeks, Singleton pregnancy, Cervical insufficiency, Emergency cervical cerclage, Adverse neonatal outcomes

## Abstract

**Background:**

Cervical cerclage is the only effective treatment for cervical insufficiency, effectively preventing late miscarriage and preterm birth. The effectiveness and safety of emergency cervical cerclage (ECC) as an emergency treatment when the cervix is already dilated or when there is protrusion of the fetal membranes into the vagina remain controversial, especially in pregnancies at 24–28 weeks when the fetus is viable. There is still no consensus on whether emergency cervical cerclage should be performed in such cases.

**Purpose:**

To investigate the effectiveness and safety of emergency cervical cerclage in singleton pregnant women at 24–28 weeks of gestation.

**Methods:**

This study employed a single-center prospective cohort design, enrolling singleton pregnant women at 24–28 weeks of gestation with ultrasound or physical examination indicating cervical dilation or even membrane protrusion. Emergency cervical cerclage was compared with conservative treatment. The primary endpoints included a comprehensive assessment of perinatal pregnancy loss, significant neonatal morbidity, and adverse neonatal outcomes. Secondary endpoints included prolonged gestational age, preterm birth, neonatal hospitalization rate, premature rupture of membranes, and intrauterine infection/chorioamnionitis.

**Results:**

From June 2021 to March 2023, a total of 133 pregnant women participated in this study, with 125 completing the trial, and were allocated to either the Emergency Cervical Cerclage (ECC) group (72 cases) or the conservative treatment group (53 cases) based on informed consent from the pregnant women. The rate of adverse neonatal outcomes was 8.33% in the ECC group and 26.42% in the conservative treatment (CT) group, with a statistically significant difference (*P* = 0.06). There were no significant differences between the two groups in terms of perinatal pregnancy loss and significant neonatal morbidity. The conservative treatment group had a mean prolonged gestational age of 63.0 (23.0, 79.5) days, while the ECC group had 84.0 (72.5, 89.0) days, with a statistically significant difference between the two groups (*P* < 0.001). Compared with CT group, the ECC group showed a significantly reduced incidence of preterm birth before 28 weeks, 32 weeks, and 34 weeks, with statistical significance (*P* = 0.046, 0.007, 0.001), as well as a significantly decreased neonatal hospitalization rate (*P* = 0.013, 0.031). Additionally, ECC treatment did not increase the risk of preterm premature rupture of membranes or intrauterine infection/chorioamnionitis, with no statistically significant differences (*P* = 0.406, 0.397).

**Conclusion:**

In singleton pregnant women with cervical insufficiency at 24–28 weeks of gestation, emergency cervical cerclage can reduce adverse neonatal pregnancy outcomes, effectively prolong gestational age, decrease preterm births before 28 weeks, 32 weeks, and 34 weeks, lower neonatal hospitalization rates, and does not increase the risk of preterm premature rupture of membranes or intrauterine infection/chorioamnionitis.

## What does this study add to the clinical work

**Table:** 

This article can provide a theoretical basis for the effectiveness and safety of emergency cervical cerclage in singleton pregnant women with cervical insufficiency at 24–28 weeks of gestation.	

## Introduction

Preterm birth is a significant contributor to global neonatal mortality and long-term health issues among surviving infants [1], with approximately 1 in 15 (1 million) children under the age of 5 worldwide dying from complications related to preterm birth each year [2]. Despite considerable advancements in neonatal intensive care technology and perinatal care over the past few decades leading to improved survival rates of extremely preterm infants, the immaturity of multiple organ systems in surviving neonates often results in physical and neurodevelopmental sequelae, impacting long-term quality of life. Therefore, the prevention of preterm birth remains central to enhancing maternal and neonatal healthcare [3]. Prophylactic cervical cerclage is an effective procedure for the treatment of cervical insufficiency with a history of indications and has been widely used in clinical practice with significant efficacy. However, the potential benefits of emergency cervical cerclage, as a rescue measure applied during gestational weeks 24–28, remain uncertain. On one hand, successful surgery may prolong gestational weeks, increase the chances of neonatal survival, and improve the quality of neonatal survival. On the other hand, the procedure may induce uterine contractions or lead to concurrent infections and preterm premature rupture of membranes, resulting in premature termination of pregnancy and affecting the prognosis of the mother and child. This study aims to explore the effectiveness and safety of emergency cervical cerclage in singleton pregnancies with cervical insufficiency at 24–28 weeks of gestation.

## Materials and methods

### Study design and participants

This single-center, open-label cohort study was conducted at Shenyang Maternal and Child Health Hospital from June 2021 to March 2023. The study enrolled singleton pregnant women at 24–28 weeks of gestation with indications including cervical dilation detected by ultrasound, with or without protrusion of the fetal membranes into the cervical canal, or cervical dilation indicated by physical examination. Exclusion criteria included twin or multiple gestations, presence of painful uterine contractions or vaginal bleeding, severe complications such as placental abruption or fetal malformations, and consideration of possible chorioamnionitis. After providing a detailed explanation of the purpose of the study and the pros and cons of the two treatment options to the pregnant women and their families, informed consent was obtained, and the pregnant women autonomously selected the treatment method before being allocated to either the emergency cervical cerclage (ECC) group or the conservative treatment (CT) group. A database was established for the study, and demographic data of the participants (such as age, history of vaginal delivery, history of induced abortion, history of hysteroscopy, etc.) were entered into the computer system. Ultrasound examinations were performed by senior attending physicians with a deputy chief physician title or above using transvaginal ultrasound, while physical examinations were conducted by obstetricians with a deputy chief physician title or above. All pregnant women signed informed consent forms to participate in this study. This study has been approved by the Ethics Committee of Shenyang Maternal and Child Health Hospital (2,02,129).The trial was registered under ChiCTR2300077797 with Chinese Clinical Trial Registry.

To maintain the vaginal environment, pregnant women with abnormal secretions underwent daily vaginal disinfection. During the treatment period, clinical physicians in both groups decided whether to use tocolytic agents and antibiotics based on the patient’s condition. Surgical procedures for the ECC group were conducted in the operating room, with patients placed in the lithotomy position under either epidural or combined spinal-epidural anesthesia administered by experienced obstetricians with the title of deputy chief physician or above. The cervical cerclage was performed using the McDonald technique, and for patients with cervical dilation and prolapsed membranes, the fetal membranes were manually replaced using a Foley catheter balloon. The suturing material used was MERSILENE tape manufactured by Johnson & Johnson. The CT group received bed rest and/or appropriate tocolytic medication. Diagnosis and treatment of other complications and comorbidities during pregnancy were performed according to clinical guidelines. Data collected included gestational age at intervention, gestational age at delivery, occurrences of preterm premature rupture of membranes (PPROM), intrauterine infection, and postpartum placental pathology examination results. Additionally, information on the survival and health status of newborns, as well as their admission to the neonatal intensive care unit after birth, was also collected.

## Research indicators

### Primary indicators

The main outcome measures for this study included perinatal pregnancy loss, significant neonatal morbidity, and adverse neonatal outcomes.

1. Perinatal pregnancy loss: this encompasses miscarriage, stillbirth, and neonatal death.
2. Significant neonatal morbidity: this refers to the occurrence of at least one of the following complications in newborns before discharge: bronchopulmonary dysplasia, periventricular leukomalacia, necrotizing enterocolitis, sepsis confirmed by blood culture, and intracranial hemorrhage (as determined by neonatologists).
3. Adverse neonatal outcomes: this includes both perinatal pregnancy loss and significant neonatal morbidity.

### Secondary indicators

Secondary outcome measures included prolonged gestational time, delivery before 28 weeks, 32 weeks, and 34 weeks, preterm premature rupture of membranes, intrauterine infection/chorioamnionitis, and neonatal hospitalization rate. Prolonged gestational time was measured in days, with gestational age calculated up to full term (≥ 37 weeks).

### Statistical analysis

Statistical analysis was conducted using SPSS 26.0. Normality and homogeneity of variance tests were performed for quantitative variables. Normally distributed data were presented as mean ± standard deviation and compared between groups using an independent samples *t*-test. Non-normally distributed data were presented as median (Q1, Q3) and compared using the Mann–Whitney *U* test. Count data were presented as *n* (%) and compared using the chi-square test or Fisher’s exact probability method. The Kaplan–Meier curve was used to describe the cumulative prolongation of gestation time, and survival curves were plotted. The Log-rank test was applied for the univariate analysis of ECC, and the Cox proportional hazards regression model was established. A *P*-value of less than 0.05 indicated statistical significance.

## Result

**Fig. 1:**
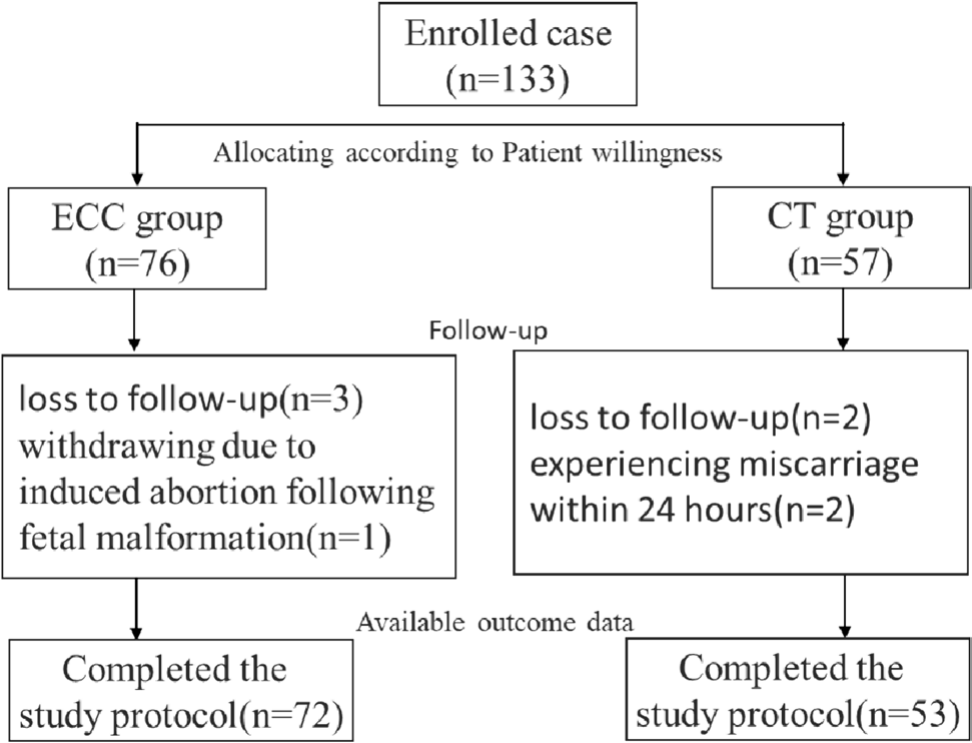
Study flow chart

The study enrolled a total of 133 pregnant women, with 76 in the ECC group and 57 in the CT group, based on the informed choice of the pregnant women. In the ECC group, 72 pregnant women completed the study, with 3 lost to follow-up and 1 withdrawing due to induced abortion following fetal malformation. In the CT group, 53 pregnant women completed the study, with 2 lost to follow-up and 2 experiencing miscarriage within 24 h. The specific research procedures are outlined in Fig. 1.

### The demographic and clinical characteristics

In the ECC group, the average gestational age Tabata intervention was 25.2 ± 0.9 weeks, while in the CT group, the average gestational age at enrollment was 25.8 ± 1.4 weeks. In terms of demographic and clinical characteristics, there were no statistically significant differences between the two groups of pregnant women. Clinical characteristics included the primary causes of cervical insufficiency (history of vaginal delivery, history of induced abortion, history of hysteroscopic surgery, and pre-existing vaginitis) and the status of cervical dilation (all *P* > 0.05), as detailed in Table 1.

**Table 1:** Demographic and clinical characteristics of pregnant women in two groups (*n* = 125)

Characteristics	ECC group (*n* = 72)	CT group (*n* = 53)	Inspection value (*t/χ*^2^)	*P* value
Age(years) ( *x* ± *s*)	31.5 ± 4.5	31.0 ± 4.0	− 0.723	0.471
Labor or not [ *n* ( %)]	6 (8.3)	4 (7.5)	0.0001	1
History of hysteroscopic surgery [ *n* ( %)]	10 (13.9)	5 (9.4)	0.574	0.449
Vaginitis [ *n* ( %)]	21 (29.2)	11 (20.8)	1.134	0.287
Cervical opening [ *n* ( %)]	21 (29.2)	8 (15.1)	3.393	0.065

### Primary indicators

In the ECC group, out of 72 pregnant women with complete data, one underwent cesarean section at 26^+3d^ weeks due to placental abruption, resulting in neonatal death after family refusal for treatment. Another case underwent cesarean section at 32^+5d^ weeks due to PPROM, and the neonate died from sepsis 2 days after birth. The perinatal loss rate was 2.78% (2/72). In the CT group, out of 53 pregnant women with complete data, five experienced miscarriage 2–15 days after tocolytic therapy, and two delivered naturally at 28^+4d^ and 29^+4d^ weeks, resulting in neonatal deaths due to sepsis and multiple organ failure. The perinatal loss rate was 13.20% (7/53). There was no statistically significant difference between the two groups (*P* = 0.06), as detailed in Table 2.

**Table 2:** Comparison of major pregnancy outcomes between the two groups (*n* = 125)

Primary indicators	ECC group (*n* = 72)	CT group (*n* = 53)	Inspection value (*χ*^2^)	*P* value
Perinatal pregnancy loss [ *n* (%)]	2 (2.78)	7 (13.20)	3.532	0.06
Significant neonatal morbidity [ *n* (%)]	4/70 (5.71)	7/46 (15.22)	1.918	0.166
Adverse neonatal outcomes [ *n* (%)]	6 (8.33)	14 (26.42)	7.426	0.006

### Secondary outcomes

The ECC group had a prolonged gestational age of 84 (72.5, 89) days, while the CT group had a prolonged gestational age of 63.0 (23.0, 79.5) days. The difference between the two groups was statistically significant (*P* < 0.001), indicating that ECC was more effective in prolonging gestational age compared to conservative treatment. The proportion of deliveries before 28 weeks, 32 weeks, and 34 weeks, as well as the neonatal hospitalization rate, were lower in the ECC group than in the CT group, and the differences between the two groups were statistically significant (*P* < 0.05), indicating that ECC could reduce the rate of preterm birth and neonatal hospitalization. Among non-miscarriage pregnant women, the incidence of PPROM in the ECC group was 12.7% (9/71), and in the CT group, it was 6.3% (3/48), with no statistically significant difference between the two groups (*P* = 0.406). In the ECC group, three cases were diagnosed with chorioamnionitis postoperatively, while no cases occurred in CT group. The ECC group did not experience other complications such as cervical lacerations. Refer to Table 3 for details. In summary, the ECC group had advantages over the CT group in terms of prolonging gestational age, reducing the rate of preterm birth, and lowering neonatal hospitalization rates, without increasing the occurrence of PPROM, chorioamnionitis, and other complications.

**Table 3:** Comparison of secondary pregnancy outcomes between two groups (*n* = 125)

Secondary outcomes	ECC group (*n* = 72)	CT group (*n* = 53)	*t /χ2*	*P* value
Prolonged gestational age (days)	76.17 ± 19.26	53.74 ± 30.04	− 4.763	< 0.001
Delivery before 28 weeks [ *n* (%)]	1 (1.4%)	6 (11.3%)	3.973	0.046
Delivery before 32 weeks [ *n* (%)]	8 (11.1%)	16/53 (30.2%)	7.162	0.007
Delivery before 34 weeks [ *n* (%)]	11 (15.2%)	22/53 (41.5%)	10.811	0.001
Neonatal hospitalization rate[ *n* (%)]	10/71 (14.08%)	16/48 (33.3%)	6.214	0.013
PPROM [ *n* (%)]	9/71 (12.7%)	3/48 (6.3%)	0.692	0.406
Chorioamnionitis [ *n* (%)]	3/71 (4.2%)	0/48 (0%)	0.716	0.397

**Fig. 2:**
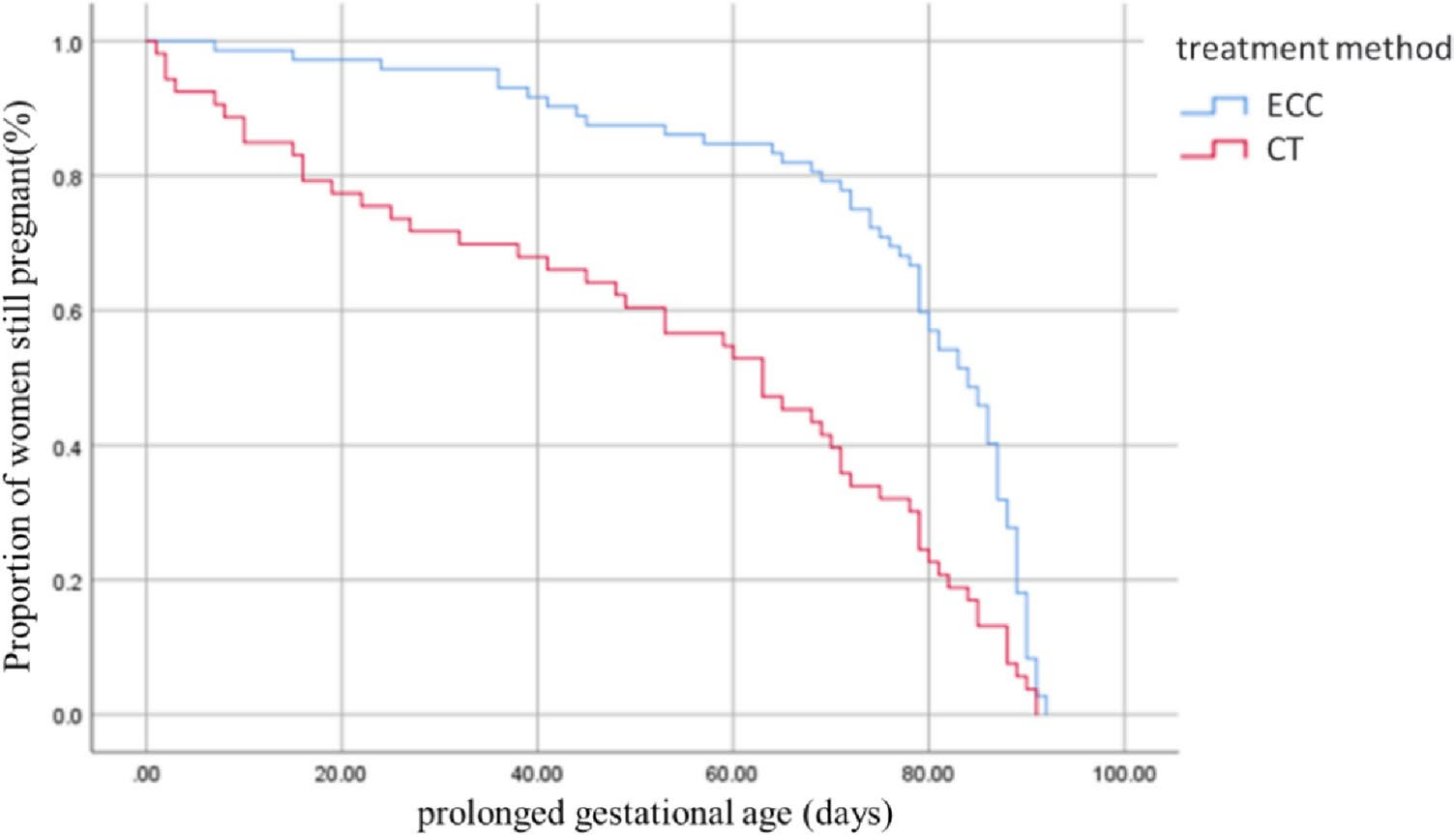
Survival curves associated with prolonged gestational days with two different treatments

The Kaplan–Meier curve depicted the cumulative distribution of prolonged gestational age, with full-term delivery defined as reaching 37 weeks of pregnancy. In the ECC group, 84.7% (61/72) of the pregnant women had a prolonged gestational age of ≥ 60 days, whereas in the CT group, only 55.8% (29/52) achieved this milestone. The log-rank univariate analysis indicated a significant difference in prolonged gestational age between the two groups (*χ*^2^ = 18.631, *P* < 0.001). The COX regression model demonstrated that ECC was a protective factor for prolonged gestational weeks, with HR = 0.477, *P* < 0.001. Refer to Fig. 2 for details.

## Discussion

Currently, the treatment options for cervical insufficiency mainly include conservative and surgical treatments. Emergency cervical cerclage is an immediate treatment for pregnant women with cervical insufficiency after cervical dilation [4]. Before 24 weeks of gestation, guidelines from various countries recommend emergency cervical cerclage to prolong gestational age and provide the fetus with a better chance of survival. However, there is no clear consensus on whether emergency cervical cerclage should be performed after 24 weeks of gestation. The Royal College of Obstetricians and Gynaecologists (RCOG) in the UK and the Queensland Clinical Guideline [5] suggest individualized decision-making. RCOG considers the high morbidity and mortality rates of extremely preterm neonates, while the Queensland Clinical Guideline is based on the limited available data supporting the effectiveness of emergency cerclage.

Due to ethical reasons, most of the data on the effectiveness of emergency cervical cerclage (ECC) comes from retrospective studies. The results show that ECC can extend pregnancy by 13.8 to 49.1 days and the neonatal survival rate fluctuates between 46 and 90% [6–9]. In 2015, a prospective study by Ciancimino et al. also confirmed the positive results of emergency cervical cerclage with 12 patients receiving ECC at 17–26 weeks of gestation, resulting in an average prolongation of pregnancy by 89.9 days and a neonatal survival rate of 83.3% [10]. Several observational studies comparing ECC with conservative treatment have demonstrated significant benefits of ECC in prolonging gestational weeks, reducing preterm delivery before 32 and 34 weeks, and improving neonatal survival rates compared to conservative treatment [11–17]. However, it is regrettable that the included literature mostly consists of small-sample retrospective studies with low data quality and high risk of bias. In order to increase the sample size and improve statistical power, in recent years, several scholars have published meta-analyses on ECC. A meta-analysis published in Obstetrics and Gynecology in 2015 summarized the literature on the use of emergency cerclage in singleton pregnancies with cervical dilation of at least 0.5 cm. The data were derived from 10 studies (including 1 randomized controlled trial, 2 prospective cohort studies, and 7 retrospective studies) involving 757 pregnant women at gestational weeks ranging from 14 to 27. Among them, 485 received ECC and 272 received conservative treatment. The results showed that compared to conservative treatment, ECC increased neonatal survival rates and extended the average gestational period by 33.98 days [18]. In 2020, Christos Chatzakis et al. published a meta-analysis summarizing and analyzing 12 studies involving 1021 pregnant women. The conclusion was that implementing emergency cervical cerclage before 28 or 32 weeks of gestation was more beneficial than expectant management in extending gestational weeks, increasing the gestational age at delivery (by > 5 weeks), reducing the risk of neonatal admission to the intensive care unit, and fetal mortality, among other aspects [19]. A latest meta-analysis published in PLoS One in 2023 also indicated that, before 28 weeks of gestation, for singleton pregnancies with cervical dilation due to cervical insufficiency, emergency cervical cerclage can significantly prolong the duration of pregnancy and improve neonatal survival rates compared to expectant management [20].

Our results differ from previous studies and meta-analyses that have indicated the efficacy of ECC in extending gestational weeks and improving neonatal survival rates. In our study, among singleton pregnancies with cervical insufficiency at gestational weeks 24–28, the implementation of emergency cervical cerclage did not reduce the rate of pregnancy loss or severe neonatal morbidity. However, it did show benefits in reducing adverse neonatal outcomes, prolonging gestational weeks, decreasing preterm birth rates before 28, 32, and 34 weeks, and lowering neonatal hospitalization rates. These findings align with the results of Alfirevic Z et al. in 2017, where their study suggested that ECC only reduced the preterm birth rate without impacting perinatal mortality and neonatal morbidity [3]. Although our study showed no statistically significant differences in the rate of pregnancy loss or severe neonatal morbidity between the ECC group and the CT group, there were substantial disparities in the data (pregnancy loss: 2.78% vs. 13.20%; severe neonatal morbidity: 5.71% vs. 15.22%). This may be attributed to advancements in neonatal care and medical technologies, enabling the survival of many extremely preterm infants, thus indicating that ECC primarily extends the duration of pregnancy without significantly improving live birth or survival rates. Moreover, the discrepancy could also stem from an insufficient number of cases in the study, leading to false negatives. This necessitates further research or multi-center studies with expanded sample sizes to obtain more reliable statistical data.

In conclusion, our study suggests that while ECC may not directly reduce the risk of pregnancy loss or severe neonatal morbidity, it does offer benefits in improving neonatal outcomes and reducing preterm birth rates. Further research, including multi-center studies with larger sample sizes, is warranted to validate and expand upon these findings.

ECC has been associated with a potential risk of membrane rupture and infection, leading to the shortening of pregnancy. The reported rates of membrane rupture have varied from 5 to 25% [21, 22]. At different stages of pregnancy, membrane rupture may lead to miscarriage or affect fetal survival to varying degrees. However, a retrospective study conducted at the Medical Center of the PLA General Hospital in Beijing, China, from January 1, 2007, to January 31, 2017, involving 50 cases of singleton pregnancies with cervical insufficiency at ≤ 28 weeks gestation, showed that no patients experienced membrane injury due to the surgery, and 5 cases (10%) developed chorioamnionitis [23]. Furthermore, a meta-analysis published in PLoS One in 2023 not only demonstrated that ECC significantly prolongs gestational time and improves neonatal survival rates before 28 weeks of pregnancy, but also found that the risk of chorioamnionitis and premature rupture of membranes during and after ECC did not differ from conservative treatment [20]. These findings are consistent with our study results, indicating that ECC does not increase the risk of PPROM or adverse perinatal outcomes. It is important to note that these results provide valuable insights into the safety and efficacy of ECC, particularly in relation to the risks of membrane-related complications, thereby contributing to the body of evidence on the comparative outcomes of ECC and conservative treatments in the management of cervical insufficiency.

In previous small-scale studies, progesterone, non-steroidal anti-inflammatory drugs (NSAIDs), and prophylactic antibiotics have been used as adjuvant therapies for ECC to varying extents. However, there is little evidence to recommend them as standalone treatments for this condition. Urinary tract infections and bacterial vaginosis can cause cervical dilation, thereby increasing the risk of preterm birth. Therefore, if a urinary tract infection or bacterial vaginosis is suspected or diagnosed, antibiotic treatment may be used. Further research on interventions for indications other than ECC has not been conducted. The decision to use progesterone, antibiotics, and tocolytics in pregnant women is based on clinical experience.

The clinical application of ECC as an emergency treatment for cervical dilation, and even prolapsed membranes into the vagina, remains controversial. Our study indicates that performing ECC at 24–28 weeks of pregnancy reduces adverse neonatal outcomes, effectively prolongs gestational time, reduces the rates of preterm births before 28, 32, and 34 weeks, and also decreases neonatal hospitalization rates. Moreover, it does not increase the risk of PPROM or intrauterine infection/chorioamnionitis. However, given that our study is from a single center with a relatively limited number of cases, we need to cautiously interpret the statistical results. Furthermore, considering the continuously improving level of neonatal care, strict patient selection and thorough communication with patients and their families are necessary before performing emergency cerclage. This includes discussing the risks and benefits of the surgery and developing individualized treatment plans, along with the judicious use of antibiotics, tocolytic agents, and other intervention therapies, aiming to extend gestational weeks and improve neonatal survival rates.

## Data Availability

The data that support the findings of this study are available from the corresponding author, [Jing Yang], upon reasonable request.
